# Ethnically distinct populations and coping with violence against children in the COVID-19 pandemic

**DOI:** 10.1590/0034-7167-2023-0350

**Published:** 2024-03-11

**Authors:** Ana Tereza Torquato de Morais Navarro, Lucimara Fabiana Fornari, Emiko Yoshikawa Egry, William Dias Borges, Rosemeire Natsuko Shoji

**Affiliations:** I Universidade de São Paulo. São Paulo, São Paulo, Brazil

**Keywords:** Child Abuse, Public Policy, Ethnicity, COVID-19, Program Evaluation, Maltrato Infantil, Políticas Públicas, Grupos Étnicos, COVID-19, Evaluación de Programas y Proyectos de Salud, Maus-Tratos Infantis, Políticas Públicas, Grupos Étnicos, COVID-19, Projetos de Saúde

## Abstract

**Objective:**

To identify policies and programs adopted by a Brazilian municipality to address violence against children during the COVID-19 pandemic.

**Method:**

A qualitative documentary study. The study setting was the municipality of Ananindeua, Pará, Brazil. Data was collected from official websites between November 2021 and February 2022. Thematic content analysis was used with the support of webQDA software.

**Results:**

Three empirical categories emerged: a) Impacts of the COVID-19 pandemic on violence against children; b) Action strategies for tackling violence against children in the COVID-19 pandemic; c) Evaluation indicators and targets for action strategies for tackling violence against children.

**Final considerations:**

The documents revealed few direct mentions of children, especially traditional populations; they presented superficial and ineffective evaluations of the policies and programs adopted, using exclusively quantitative indicators.

## RESUMO

**Objetivo:**

Identificar políticas e programas adotados por um município brasileiro para o enfrentamento da violência contra crianças durante a pandemia de COVID-19.

**Método:**

Estudo qualitativo do tipo documental. O cenário de estudo foi Ananindeua, Pará, Brasil. A coleta de dados foi realizada em sites oficiais, de novembro de 2021 a fevereiro de 2022. Utilizou-se a análise de conteúdo temática apoiada pelo *software* webQDA.

**Resultados:**

Emergiram três categorias empíricas: a) Impactos da pandemia de COVID-19 na violência contra crianças; b) Estratégias de enfrentamento desta violência; c) Indicadores de avaliação e metas das estratégias para este enfrentamento

**Considerações finais:**

Há poucas menções diretas às crianças, especialmente às de populações tradicionais; apresentaram avaliações ineficazes das políticas, apenas qualitativamente.

## RESUMEN

**Objetivo:**

Identificar políticas y programas adoptados por un municipio brasileño para hacer frente a la violencia infantil durante la pandemia de COVID-19.

**Método:**

Se trata de un estudio cualitativo documental. El escenario fue el municipio de Ananindeua, Pará, Brasil. Los datos se recogieron de sitios de internet oficiales entre noviembre de 2021 y febrero de 2022. Se utilizó el análisis temático de contenido con el apoyo del software webQDA.

**Resultados:**

Surgieron tres categorías empíricas: a) Impactos de la pandemia de COVID-19 en la violencia contra los niños; b) Estrategias de acción para abordar esta violencia; c) Indicadores de evaluación y metas de las estrategias de acción para este afrontamiento.

**Consideraciones finales:**

Los documentos revelaron pocas menciones sobre los niños de las poblaciones tradicionales en especial y presentaron evaluaciones superficiales de las políticas y programas adoptados, utilizando exclusivamente indicadores cuantitativos.

## INTRODUCTION

Violence is a social and historical construction, which can increase or decrease according to its reproduction in society at the collective and individual levels^(1)^. It is a complex phenomenon with multiple causes. Among its manifestations are structural, social and interpersonal violence^(2)^, in which children are exposed to different vulnerabilities.

Designing public policies aimed at ethnic groups is challenging due to the heterogeneity of these populations, who suffer from social invisibility and structural prejudice. In recent years, support services for ethnic groups have aimed to qualify humanized care in order to deal with language difficulties, access to traditional means of communication or understanding of national legislation. In addition, the COVID-19 pandemic has seen changes in the social dynamics of circulation, so that diverse cultures and the contexts in which they are inserted imply different and unequal access to rights^(3)^.

During the most critical period of the COVID-19 pandemic, there were rules in place to reduce the transmission of the disease, such as social distancing, including the temporary closure of schools and daycare centers, the main support services for dealing with violence against children. This measure has also altered social routines and relationships, resulting in children spending more time at home, as well as the need for comprehensive care by guardians.

The World Health Organization (WHO), in a publication with End Violence, called on governments and the international community to protect children from the risks of violence through a collective response, including mental health and psychosocial support, social protection and care for the most vulnerable children and in foster institutions^(4)^.

Although there is no estimate in Brazil of how much the pandemic has affected migrants and refugees, this group is characterized as one of the most impacted by COVID-19. Numerous migrants, who were already in poverty beforehand, were unable to get emergency aid or support from the Brazilian state^(5)^.

Study by Shimabukuro et al.^(6)^ which looked at policies and programs to combat violence in a municipality in the southern region of Brazil, found that one of the main impacts was the paradox between the need for social distancing to contain the pandemic and the consequent exposure of victims to aggressors in the domestic space, with a decrease in notifications and complaints.

The impact of COVID-19 on the population of migrants and refugees, Indigenous people and quilombolas has been alarming during this period^(5)^. In this context, the municipality of Ananindeua, Pará, was selected for the study due to its ethnic diversity and processes of vulnerability. This study is a continuation of the aforementioned work, which aimed to contribute to the debate on policies and programs to tackle violence against children in situations of exceptional confinement at home, where the perpetrators of this violence are mostly present.

## OBJECTIVE

To identify the policies and programs of a Brazilian municipality to deal with violence against children during the COVID-19 pandemic.

## METHODS

### Ethical aspects

Although the present study does not directly address human beings due to its documentary nature, the main project to which it is linked, Good Nursing Practices in Health Care for Traditional Peoples, is approved by the Research Ethics Committee of the School of Nursing of the University of São Paulo.

### Theoretical-methodological framework

This is a qualitative documentary study based on the Theory of Practical Intervention in Public Health Nursing ( TIPESC)^(6)^. TIPESC was built to understand the contradictions of the objective reality of Public Health Nursing as a field of theory and practice. It is a nursing theory based on a historical and dialectical materialist worldview, which seeks nursing intervention through a dynamic, dialectical and participatory methodology^(7)^.

Considering the diversity of qualitative research, we opted for the documentary type given the nature of the phenomenon and subject of this study. Documentary research uses materials from various sources without prior analytical treatment. It differs from bibliographical research in that it uses scientific studies that have been analyzed as sources^(8)^.

In documentary research, the choice of document to be analyzed is not random and depends on the phenomenon and the research question to be answered based on a given theoretical framework^(8)^. Therefore, for this study, the documents chosen were the policies and programs relating to tackling violence against children in the municipality of Ananindeua during the COVID-19 pandemic.

### Kind of study

It is a qualitative documentary study. In the documentary research method, the researcher engages in the study domain, seeking to apprehend the phenomenon through the perspectives presented in the documents. Documentary research is one in which data is exclusively obtained from documents, with the purpose of extracting information contained in them, in order to comprehend a specific scientific inquiry. In this type of research, the goal is to immerse oneself in documents with the aim of extracting research data from them, and after analysis, achieve a broader understanding of a given phenomenon^(9,10)^.

Documents published in secondary sources were utilized, including those at the municipal level (Ananindeua, Pará), state level (State of Pará), and federal level (Brazil), for the selected and analyzed documents.

As means of standardizing qualitative studies, the Standards for Reporting Qualitative Research (SRQR) instrument was employed to describe the methodological procedures. However, given the qualitative documentary nature of the study, it does not contain data on research subjects; hence, the methodology does not fully encompass all items outlined in the SRQR.

### Study Setting

The municipality of Ananindeua is located in the metropolitan region of Belém, in the state of Pará, Brazil. Its estimated population in 2019 was 471,980. According to the last demographic census carried out in 2010, it has a life expectancy at birth of 74.24 years, a Human Development Index of 0.718 and a GINI Index, which measures social inequality, of 0.520^(11)^.

Part of Ananindeua’s population is made up of traditional peoples and communities such as riverside communities, quilombolas and Venezuelan Indigenous peoples in humanitarian refuge, especially the Warao ethnic group, as well as other Brazilian Indigenous peoples in an urban context due to issues related to education and health.

### Data source

The consulted electronic pages were: Municipal Government of Ananindeua (PMA), Public Ministry of Pará (MPPA), State Government of Pará (PARÁ), Public Defender’s Office of the State of Pará (DPE-PA), Federal Government (BRASIL), National Council of Justice (CNJ), Chamber of Deputies (C MARA), National Council for the Rights of Children and Adolescents (CONANDA), National Health Council (CNS), United Nations International Children’s Emergency Fund (UNICEF), Ministry of Women, Family, and Human Rights (MDH), Federal Senate (SENADO), and United Nations High Commissioner for Refugees (ACNUR).

### Data Collection and Organization

The policies and programs were accessed in the search field of the websites investigated, using the terms: domestic violence, violence against children, pandemic, policies and programs. All the information obtained was read, taking into account the eligibility criteria of the documents and then selecting those that would form part of the study.

The searches were carried out between November 2021 and February 2022. The time frame corresponded to the months of March 2020 to December 2021, with the former referring to the beginning of the health crisis and the latter to the end of the current year.

The criteria for including the documents were: policies or programs covering the municipality of Ananindeua on the subject of tackling violence against children; measures to contain violence against children in the context of the COVID-19 pandemic; state and national policies or programs that support or are related to Ananindeua’s policies and programs on the subject under investigation. The exclusion criteria were: documents, programs or policies that were not published during the COVID-19 pandemic during the research period.

The data was extracted using a semi-structured instrument containing the title of the document, source, type of document, year of publication, sector of coverage, target audience, understanding of violence, objective of the policy or program, action strategies, goals, forms of evaluation and recommendations for tackling violence against children adapted for the Microsoft Excel® spreadsheet.

### Data analysis

This spreadsheet was inserted into the webQDA qualitative analysis software using the automatic import tool. The documents were then analyzed using the thematic content analysis technique, which includes the stages of pre-analysis, exploration of the material, treatment of the results, interpretation and inference^(12)^. The data corresponding to the characterization of the documents was automatically coded as Descriptors (source, type of document, year of publication, sector covered and target audience). The other information associated with the empirical data (understanding of violence, objective of the policy or program, action strategies, goals, forms of evaluation and recommendations for tackling violence against children), imported into the Internal Sources system, was categorized using the Coding system, using the Tree Codes tool. The first author of the study coded all the documents and the other authors validated them using the hidden coding tool.

## RESULTS

The final study sample consisted of 22 documents. Most of the documents were national (n=14), followed by state (n=6) and municipal (n=2). With regard to the year of publication, 14 documents were published in 2021 and eight documents in 2020, showing an increase in the number of publications in the second year of the pandemic. As for the types of documents found, recommendations (n=4), manuals (n=3), programs (n=3), decree, law, administrative procedure, Municipal Plan, State Plan, project, commission, agreement, campaign, guide, booklet and protocol (n=1) were selected. Chart 1 shows all the document types.

**Chart 1:** Policies and programs to address violence against children reported in Ananindeua during the COVID-19 pandemic

Documents		
Document number	Territorial coverage	Document title
1	Ananindeua	Establishes the Municipal Intersectoral Committee of principles and guidelines for the elaboration and implementation of Public Policies for Early Childhood in the Municipality of Ananindeua in the State of Para and makes other provisions^(13)^
2	Ananindeua	Provides for the nullity of the appointment or hiring, for certain public positions and jobs, of a person convicted of a sexual crime against a child or adolescent in the municipality of Ananindeua, and makes other provisions^(14)^
3	Para	Post-COVID Children’s Program has provided more than 10,000 consultations at the Metropolitan Polyclinic^(15)^
4	Para	The aim is to monitor and supervise the continuity of services provided by the municipal bodies in the network for protecting the rights of children and adolescents in the municipality of Limoeiro do Ajurú (Guardianship Council, CREAS and CRAS)^(16)^
5	Para	MPPA participates in the launch of the Municipal Plan to Combat Violence against Children and Adolescents^(17)^
6	Para	Para State Plan to Combat Sexual Violence against Children and Adolescents, approved by CEDCA Resolution 083/2021^(18)^
7	Para	MPPA participates in the presentation of the “Awuré Ubuntu” project. Project aims to implement public policies to combat violence against children and adolescents^(19)^
8	Para	MPPA participates in the installation of Alepa’s Early Childhood Commission^(20)^
9	Brazil	UNICEF signs agreement with MMFDH to create app to protect children and adolescents from violence^(21)^
10	Brazil	Document of the UNICEF Cooperation Program with Brazil for the period 2017-2021^(22)^
11	Brazil	Establishes the National Program to Combat Violence against Children and Adolescents and the Intersectoral Commission to Combat Violence against Children and Adolescents^(23)^
12	Brazil	CONANDA recommendations for the comprehensive protection of children and adolescents during the COVID-19 pandemic^(24)^
13	Brazil	Recommendations on the use of resources from the fund for the rights of children and adolescents in actions to prevent the social impact of COVID-19^(25)^
14	Brazil	Recommends actions for the effectiveness of CONANDA Resolution 181/2016 during the COVID-19 pandemic^(26)^
15	Brazil	CONANDA’s recommendation for the full protection of children and adolescents when they return to school in person during the COVID-19 pandemic^(27)^
16	Brazil	Manual for the Special Testimony of Children and Adolescents Belonging to Traditional Peoples and Communities^(28)^
17	Brazil	Protect Me: Justice launches campaign to combat child violence^(29)^
18	Brazil	Community Protection Guide for Indigenous Refugees and Immigrants^(30)^
19	Brazil	Mental and psychosocial health in the COVID-19 pandemic: domestic and family violence in COVID-19^(31)^
20	Brazil	COVID-19 and child and adolescent health^(32)^
21	Brazil	Preparing schools for the return to school: a look at children and adolescents who are victims of violence^(33)^
22	Brazil	Brazilian protocol for forensic interviews with child and adolescent victims or witnesses of violence^(34)^

Source: Spreadsheet containing data on Policies and Programs addressing violence against children in the context of Ananindeua, Pará, Brazil^a^.

Based on the analysis of the documents, three empirical categories were identified: a) Impacts of the COVID-19 pandemic on violence against children; b) Action strategies for tackling violence against children during the COVID-19 pandemic; c) Evaluation indicators and targets for action strategies for tackling violence against children.

### Impact of the COVID-19 pandemic on violence against children

These documents highlight the role of the state and the family in protecting children and meeting their social needs.

*The Statute of the Child and Adolescent* (ECA) *adopts the principle of comprehensive protection, including preventive, protective and socio-educational aspects, as well as prioritizing meeting the social needs of the family of origin, so that they can strengthen themselves or acquire the conditions to care for their children in a dignified manner.* (Document n° 7)

Among the situations that require the protection of children, the documents highlight domestic and intra-family violence, which has been accentuated during the COVID-19 pandemic due to social distancing measures.

*The place where children experience the most violence is inside the home. Evidence shows that violence and children’s vulnerability increase during school closures associated with complex emergencies, which are close to the characteristics of the COVID-19 pandemic.* (Document n°12)

### Action strategies for tackling violence against children during the COVID-19 pandemic

In view of the increase in violence against children during the COVID-19 pandemic, the documents highlight as action strategies for tackling the problem the consolidation of the protection network, involving the social assistance, justice, health, education and public security sectors, as well as the strengthening of intersectoral care.

*Continuity of the services of the child and adolescent protection network, carried out by CREAS, CRAS and TUTELARY COUNCILS, in an integrated, efficient and continuous manner, including during the period of the pandemic caused by COVID-19.* (Document 2)

*The creation of committees made up of teachers, staff and managers should be encouraged, where identified and/or reported cases of violence can be discussed, spaces where these professionals can welcome each other and seek guidance when necessary. These committees should include the participation of student unions, spaces for youth representation and participation in school decision-making processes.* (Document 8)

The documents also emphasize the need to train the professionals who work in the services that make up the protection network.

*Hold training and technical guidance workshops on Sexual Violence against Children and Adolescents for civil servants in the municipalities of the state of Para who work in highly complex reception units.* (Document 5)

Another strategy widely cited in the documents for tackling the problem was the management of data on violence against children, with the aim of mapping cases and providing a basis for proposing intervention actions, especially in relation to sexual violence.

However, it was educational campaigns that stood out as the main strategies for preventing the problem. Despite this, the documents did not detail the content and target audience of these campaigns.

With regard to the ethnic-racial approach, only two of the 22 documents analyzed (Documents 16 and 22) described specific coping strategies for children belonging to traditional peoples and refugees in Brazil. These documents addressed the proposal of a special testimony protocol with a single collec tion with intercultural contours considering ethnic-social differences (Document 16), and the proposal to form a Working Group that considers gender, generation and diversity, as well as social protection strategies for short or prolonged stays in cities, especially for Indigenous people (Document 22).

### Evaluation indicators and targets for action strategies to tackle violence against children

The evaluation indicators were related to the number of services provided by the protection network, the number of training courses involving the professionals responsible for the services, the number of educational campaigns carried out mainly in schools and on social networks, as well as the number of public policies created in the municipalities that make up the state of Para.

*Number of people assisted; Number of families assisted.* (Document 7)

*Number of continuing training courses held; Number of participants in continuing training courses.* (Document 9)

*Number of thematic cultural actions and productions produced in the 12 integration regions.* (Document 8)

In addition, the evaluation indicators were associated with the process of implementing the actions proposed by the policies and programs, the adherence of the municipalities and the state of Para to the actions, and the continuous monitoring of the actions implemented.

With regard to the goals of the action strategies, the documents addressed the scope of the actions according to the number of municipalities and regions in the state of Para, the number of institutions and participants involved in the actions proposed by the policies and programs.

## DISCUSSION

An analysis of the documents revealed the prevalence of evaluation indicators at state level, as well as the absence of qualitative indicators.

With regard to the relevance of policies and programs focusing on race and ethnicity, the data shows that little importance has been given to this social category, making this group invisible in the eyes of public policies for tackling violence against children. Note should be made of Document 16 on recommendations for special listening to children belonging to traditional peoples and communities. Although the manual’s recommendations are pertinent, with a coherent slant towards the development of cultural competence^(35)^, These only cover the legal aspect of listening. Thus, in general, there is a lack of policies and programs that anticipate the perpetration of violence and can help prevent it, especially in traditional populations.

Regarding the empirical categories that emerged from the thematic analysis with the support of webQDA, the category “Impact of the COVID-19 pandemic on violence against children’, social distancing with the consequent closure of schools and confinement at home, were conditions in which the victim contradictorily spent more time living with potential aggressors. In this sense, the study by Thomas et al^(36)^ states that the COVID-19 pandemic has seen social distancing as the main measure to contain transmission of the virus. Thus, this measure was responsible for increasing the burden of emotional stress in the home due to the longer time family members spent together and the overload of caring for children^(36)^. In addition, children staying in the home increased the chances of them being trapped with their aggressors and decreased notification rates^(37)^.

In the US, a study looking at child abuse and neglect response in the time of COVID-19 found that reports of child abuse or neglect are made by people in the education sector, making educators recognized as the main reporters. Despite evidence that cases of violence against children had increased, in the initial third of the pandemic there was a decrease in reports. Unfortunately, this was a reflection of the reduction in children’s contact with educators and community youth programs^(36)^.

In Brazil, in March 2020, reports of sexual violence against children increased by 85% compared to the same period in the previous year^(15)^. Also, according to the WHO alert, the frequency of emotional or psychological abuse increased from 2.52% before the pandemic to 7% after the pandemic^(37)^.

Being subjected to silenced violence at home is a detriment to children’s health, reinforced by the need for social isolation recommended in the context of the COVID-19 pandemic. Children’s support networks have been confronted with stressors such as the economic crisis, unemployment, coping with illness and bereavement, aggravating cases where they were already suffering abuse before the COVID-19 pandemic^(38)^.

With regard to the category “Action strategies for tackling violence against children during the COVID-19 pandemic”, despite showing some intersectoral initiatives on the problem in question, the documents analyzed showed that in few cases is the leading role of children fully respected.

Qvortrup^(39)^ argues that society must be permanently attentive to the consequences of public policies for children. This justifies the present study, in that it documented policies aimed at children in situations of violence in the context of the COVID-19 pandemic, considering the vulnerable social groups highlighted in a Brazilian municipality.

Vulnerability related to violence against children needs to be analyzed and addressed at the interface between the individual and collective dimensions. This highlights the state’s responsibility for the problem and the need for debates in all social spaces to tackle it^(40)^.

From this perspective, in addition to the vulnerability related to violence against children, there is the intersectional aspect of ethnicity, which refers mainly to traditional peoples (Indigenous, quilombolas and riverside dwellers) and refugees in Brazil. In the state of Para, 546 Indigenous lands were identified in 2019, with an approximate population of 27,116 Indigenous people, representing the third state with the highest number of Indigenous people in Brazil^(11)^. In the municipality of Ananindeua, 778 people declared themselves Indigenous in 2010^(11)^.

Corroborating the results seen in Brazil, a systematic scoping review in Australia showed that experiences of abuse and neglect transcend individual trauma and include intergenerational pain and suffering in traditional peoples^(41)^, In other words, if one generation is affected, this violence impacts the generations that live with it and become aware of the violence.

Thus, proposing action strategies to tackle the problem in the municipality of Ananindeua has become essential during the COVID-19 pandemic. Based on the documentary analysis, we observed actions aimed at training human resources (28 mentions), information management (17 mentions), information campaigns (17 mentions), mapping and monitoring of cases (eight mentions), intervention programs and projects (eight mentions), notification of cases (five mentions) and digital applications for reporting and guidance (two mentions).

In addition, the documents presented actions aimed at strengthening the intersectoral network to assist children in situations of violence. Most of the documents referred to multi-professional and interdisciplinary care (43 mentions), while the other documents referred in isolation to areas such as: social assistance (17 mentions), justice (13 mentions), health (10 mentions), education (10 mentions) and public security (five mentions).

Macedo and Egry^(42)^ pointed out that multi-professional and interdisciplinary work makes it easier to tackle the problem. However, the consolidation of integrated work has yet to be achieved. This aspect was also portrayed in a Brazilian study carried out with professionals from a Specialized Social Assistance Reference Centre, which found that although the professionals recognized the logistics of the protection network and its importance in stopping violence against children and adolescents, they stressed the difficulty of protection due to the lack of structure and discontinuity of care due to a lack of human resources^(43)^.

During the COVID-19 pandemic, it was found that remote care and monitoring were actions taken to tackle violence against children^(35)^. However, this aspect was not identified in the documents analyzed. In the context of the pandemic, telephone lines were also highlighted in order to maintain communication with victimized children^(43)^.

With regard to the category “Evaluation indicators and targets for action strategies to combat violence against children”, the documents analyzed in this study revealed few direct mentions of children, especially at municipal level, and an exclusively quantitative evaluation of the indicators and targets proposed for each strategy to combat violence against children. This result highlights the lack of indicators on the quality of the actions implemented, on how they qualify care services and not necessarily just add them up, since the quantity does not reflect which of these actions are actually showing significant results. In this way, the fact that actions are only evaluated quantitatively is seen as a limiting factor, while there is a need for qualitative evaluation.

Thus, the approach to the phenomenon studied in this research, by adopting a qualitative approach through a powerful methodology and the use of software, made it possible to unveil the different perspectives of public policies for tackling violence against children, with an emphasis on ethnicity. Based on the analysis of the data, it showed that even taking into account an object of study that is complex, changing and linked to the different dimensions of objective reality, there is a reductionist tendency to focus on quantity, especially when it comes to targets and evaluating the impact of policies. Research and researchers need to overcome the mediocrity of quantifying what cannot be quantified and build goals and instruments for evaluating programs and policies based on qualitative parameters. And one of the most interesting and important issues in this respect is to assess the participation of the target public, to understand their demands and health needs from the point of view of this social group in terms of ethnicity, gender and generation.

### Contributions to the field of Nursing, Health or Public Policy

The results of the study contribute to expanding knowledge on how to deal with violence against children in a given historical moment and geopolitical space. It is recognized that measures to contain the COVID-19 pandemic have had an impact on the number of children in situations of violence. However, it is understood that the action strategies proposed through public policies and programs have proved to be limited, as they have only been evaluated using quantitative indicators. Therefore, in addition to identifying situations of violence, it is essential to critically analyze the implementation of actions to tackle the problem in concrete reality.

### Study limitations

The limitation of this study was the use of documents published and available in open access, since it did not request direct authorization from the government agencies of Ananindeua and Para to use private documents. However, this limitation does not invalidate the results identified through the study, as they provide an overview of public policies and government programs published and disseminated to address violence against children during the COVID-19 pandemic, especially the little emphasis given to ethnically diverse peoples.

## FINAL CONSIDERATIONS

The documents revealed few direct mentions of children, especially those from traditional and ethnically distinct populations, especially at the municipal level, as well as superficial and ineffective evaluations of the policies and programs adopted, using exclusively quantitative indicators, therefore limited by the lack of qualitative indicators.

The results of the study highlight the need to develop more specific policies for ethnically differentiated populations in Brazil, especially with regard to tackling violence against children, regardless of the COVID-19 pandemic. The results also reinforce the importance of new research covering situations of violence from the perspective of health work and monitoring public policies related to this important social phenomenon that interferes with children’s ways of living, getting sick and dying.

## Data Availability

^a^Borges WD, De Morais Navarro ATT, Fornari LF, Egry EY, Shoji RN. Spreadsheet with data on Policies and programs addressing violence against children in the context of Ananindeua-Pará. [Internet]. Zenodo; 2023. Available from: https://zenodo.org/record/8331332
